# Safety and efficacy of mesenchymal stem cells therapy in the treatment of rheumatoid arthritis disease: A systematic review and meta-analysis of clinical trials

**DOI:** 10.1371/journal.pone.0284828

**Published:** 2023-07-27

**Authors:** Luz Estella Mesa, Josue Guillermo López, Lucas López Quiceno, Freddy Barrios Arroyave, Karolynn Halpert, Jhyld C. Camacho

**Affiliations:** 1 BioXcellerator / BioXscience Advanced Therapies and Translational Medicine, Medellín, Antioquia, Colombia; 2 Medicarte, Armenia, Colombia; Osaka Metropolitan University, JAPAN

## Abstract

**Background and objectives:**

Some patients have insufficient treatment response to conventional disease-modifying antirheumatic drugs (cDMARD); although biologics have proven to be an effective treatment for RA, the effects that bDMARDs have on integumentary, cardiac, and immune systems and the high costs associated with these treatments, make that mesenchymal stem cell-based therapies (MSCs) for RA are being considered potential treatment methods. This work analyses the performance in safety and efficacy terms of MSCs techniques.

**Methods and finding:**

A literature search was performed in PubMed, EMBASE, Cochrane Library, Web of Science, and Open Grey databases from inception to October 28, 2022. Three randomized controlled trials (RCTs) and one non-randomized controlled trial (non-RCTs), including 358 patients met our inclusion criteria and were included in qualitative synthesis; only RCTs were eligible for quantitative synthesis (meta-analysis). Meta-analysis of adverse events (AE) in RCTs showed no significant differences in the incidence of AE in the MSCs group compared to the control group (Risk ratio: 2.35; 95% CI, 0.58 to 9.58; I^2^ = 58.80%). The pooled Risk ratio for non-serious and serious adverse events showed no statistical difference between intervention and control groups concerning the incidence of non-serious and serious adverse events (Risk ratio: 2.35; 95% CI, 0.58 to 9.51; I^2^ = 58.62%) and (Risk ratio: 1.10; 95% CI, 0.15 to 7.97; I^2^ = 0.0%) respectively. The Health Assessment Questionnaire (HAQ) and Disease Activity Score (DAS28) decreased in agreement with the decreasing values of C-reactive protein (CRP) and Erythrocyte sedimentation rate (ESR). Additionally, a trend toward clinical efficacy was observed; however, this improvement was not shown in the studies after 12 months of follow-up without continuous treatment administration.

**Conclusion:**

This Systematic review and meta-analysis showed a favorable safety profile, without life-threatening events in subjects with RA, and a trend toward clinical efficacy that must be confirmed through high-quality RCTs, considerable sample size, and extended follow-up in subjects with RA.

## Introduction

Rheumatoid arthritis (RA) is a chronic, inflammatory, and progressive autoimmune disease characterized by synovitis, destructive arthropathy, and systemic complications [1–4]. The main symptoms are joint pain, swelling, joint stiffness, malformation, loss of function, and deteriorating disability over time [5, 6]. Risk factors for developing RA are genetic and environmental [2].

According to the evidence, the prevalence of rheumatic diseases such as RA has increased in the last decades, with a global prevalence of 460 per 100.000 population between 1980 and 2019 [7–9]. Women are affected three times more than men [1]. In addition, RA is associated with increased chronic disability, morbidity, and mortality [7, 8, 10].

RA patients require long-term treatment, and according to the evidence, currently available drugs have limited efficacy [1, 5]; in fact, some patients do not achieve remission and disease control despite being treated with multiple conventional disease-modifying antirheumatic drugs (DMARDs) alone and in combination [10, 11]. Consequently, persistent inflammation leads to progressive joint and end-organ damage; therefore, the prognosis of these patients is poor and evidence the need for new therapeutic options [10].

Mesenchymal stem cells (MSCs) are multipotent progenitor cells derived from bone marrow, adipose tissue, placenta, umbilical cord, and others. MSCs are characterized by their regenerative and differentiation properties in multiple tissues, such as cartilage, muscle, tendon/ligament, and bone. Furthermore, MSCs have anti-inflammatory and immunomodulatory effects that can contribute to the therapeutic impact on several diseases, as they inhibit the proliferation and modulate immune cell responses such as T cells, B cells, dendritic cells, and natural killer cells [2–6, 11].

The immunomodulatory effect of MSCs could restore immunotolerance in RA patients, allowing complete discontinuation of immunosuppressive therapy [4]. Additionally, since the critical factor in the pathogenesis of RA is elevated cytokines that arise from numerous synovial cells, MSCs can express various receptors for pro-inflammatory cytokines to reduce inflammation in these patients [5].

In recent years, clinical studies have demonstrated the benefits of MSCs for many autoimmune diseases, including rheumatic conditions such as RA disease [5, 10]. This therapeutic potential of MSCs introduced them as an innovative treatment option for RA patients [2, 3, 12]. In this systematic review and meta-analysis, we aimed to determine the safety and efficacy of MSCs in RA patients based on available evidence from clinical trials. We statistically combined the quantitative results of the included studies to provide a more precise effect measure of the results.

## Materials and methods

The review protocol was registered on PROSPERO 2022 [CRD42022372228], available from: https://www.crd.york.ac.uk/prospero/display_record.php?ID=CRD42022372228. This systematic review was performed based on Preferred Reporting Items for Systematic Reviews and Meta-Analyses (PRISMA) recommendations.

### Search strategy

We searched PubMed, EMBASE, Cochrane Library, Web of Science databases, and the system for information on grey literature—Open Grey. The following parameters were used in the included medical electronic databases, these parameters and restrictions applied were the same in all databases: ((arthritis rheumatoid [title/abstract]) OR (rheumatoid arthritis [title/abstract]) OR (anti-citrullinated protein antibodies [title/abstract]) OR (ACPAs [title/abstract]) OR (rheumatoid factor [title/abstract])) AND ((stem cell transplantation [title/abstract]) OR (stem cells [title/abstract]) OR (stem cell research [title/abstract]) OR (cell therapy [title/abstract]) OR (cell based therapy [title/abstract]) OR (cell-based therapy [title/abstract]) OR (stem cell transplantation [title/abstract]) OR (multipotent stem cells [title/abstract]) OR (multipotent stromal cells [title/abstract]) OR (mesenchymal stem cells [title/abstract]) OR (mesenchymal stromal cells [title/abstract]) OR (mesenchymal progenitor cells [title/abstract]) OR (wharton jelly cells [title/abstract]) OR (wharton’s jelly cells [title/abstract]) OR (umbilical cord cell* [title/abstract]) OR (MSC [title/abstract]) OR (MSCs [title/abstract]) OR (ADMSC [title/abstract]) OR (ADMSCs [title/abstract]) OR (BM-MSC [title/abstract]) OR (BM-MSCs [title/abstract]) OR (BMD-MSC [title/abstract]) OR (BMD-MSCs [title/abstract]) OR (BMDMSC [title/abstract]) OR (BMDMSCs [title/abstract]) NOT (hematopoietic stem cells [title/abstract]) NOT (mononuclear [title/abstract])) AND ((clinical [title/abstract] AND trial [title/abstract]) OR (clinical trials as topic [title/abstract]) OR (clinical trial [title/abstract]) OR (random* [title/abstract]) OR (random allocation [title/abstract]) OR (placebo [title/abstract]) OR (therapeutic use [title/abstract])) AND ((humans [title/abstract]) OR (patients [title/abstract])). These records were recovered from the creation of these databases until October 28, 2022, and were limited to human studies and the English language. The search strategy for each database consulted is included as supporting information files (S2 File Search Strategy).

### Article selection

First, duplicate records were removed before the screening. Two reviewers initially screened all articles by title and abstract to exclude articles unrelated to the main objective of this study. Then, two reviewers read the full manuscripts and selected the eligible ones; a third reviewer resolved any discrepancies that arose.

### Inclusion and exclusion criteria

We selected all randomized controlled trials (RCTs) or non-randomized controlled trials (non-RCTs) that met the following criteria: (i) patients > 18 years with a diagnosis of rheumatoid arthritis (RA) according to the classification criteria ACR (American College of Rheumatology) or EULAR (European League Against Rheumatism). (ii) patients with naive RA or refractory to first- or second-line treatment. (iii) studies involving mesenchymal stem cells therapy (MSCs), autologous or allogeneic, from any source (derived from BM-MSCs bone marrow; AD-MSCs adipose tissue; UC-MSCs umbilical cord; gingival, placenta, menstrual tissue) systemic or local administration. (iv) studies that include comparison groups with cell therapy (from different sources of origin or dosage), placebo, or standard therapy. (v) randomized controlled trials evaluating the efficacy and safety of mesenchymal stem cell therapy (MSC). (vi) studies conducted in the human population and published in English. Exclusion criteria were: (i) observational (analytical or descriptive) studies. (ii) studies without a control group or placebo. (iii) studies conducted on animals, children, or pregnant patients. (iv) studies without definitive results, unavailable full text, abstract of conferences, and editorial letters.

### Data extraction and quality/risk of bias assessment tools

Two independent reviewers extracted the data from all the articles according to the following information: The first author, published year, location, trial registration number, study type, study phase, study population, age, sex, sample size, infusion route, intervention, control, follow-up, efficacy outcomes, safety outcomes; all data were extracted on a predefined form and supervised by the primary author. After data extraction, we assessed the methodological quality or risk of bias in RCTs through version 2 of the Cochrane risk-of-bias tool (RoB 2), where judgment can be "Low" or "High" risk of bias or can express "Some concerns." Moreover, we judge the risk of bias in non-RCTs through the ROBINS-I tool.

### Outcomes

#### 1. Primary outcomes

Primary outcomes were an assessment of clinical activity through DAS-28 (disease activity score 28), HAQ (health assessment questionnaire), a measure of response: measured by the ACR scale 20, 50, 70 (American College of Rheumatology 20/50/70 criteria), and serology such as anti-CCP (anti-citrullinated protein antibodies), ESR (erythrocyte sedimentation rate), CRP (C-reactive protein).

#### 2. Secondary outcomes

Secondary outcomes were the safety of treatment with MSCs: adverse events (AE), serious adverse events (SAE), and non-serious adverse events.

### Statistical analysis

A meta-analysis of the data from 3 included studies was performed by Stata 17—multivariate meta-analysis software (StataCorp, Texas, USA). Risk ratio with their corresponding 95% confidence intervals (CIs) was used for dichotomous outcome pooling. We chose a random effects model in the analysis using the DerSimonian-Laird method with the correction of zero-count cells if I^2^ is over 50% (high heterogeneity); otherwise, the fixed effects model was used. Inconsistency across studies was measured using I^2^ and a cut-off of 10% for significance. Also, we use of L’Abbé plot to investigate the potential sources of heterogeneity in the meta-analysis. Publication bias was evaluated from a funnel plot.

## Results

### Literature search

**Fig 1:**
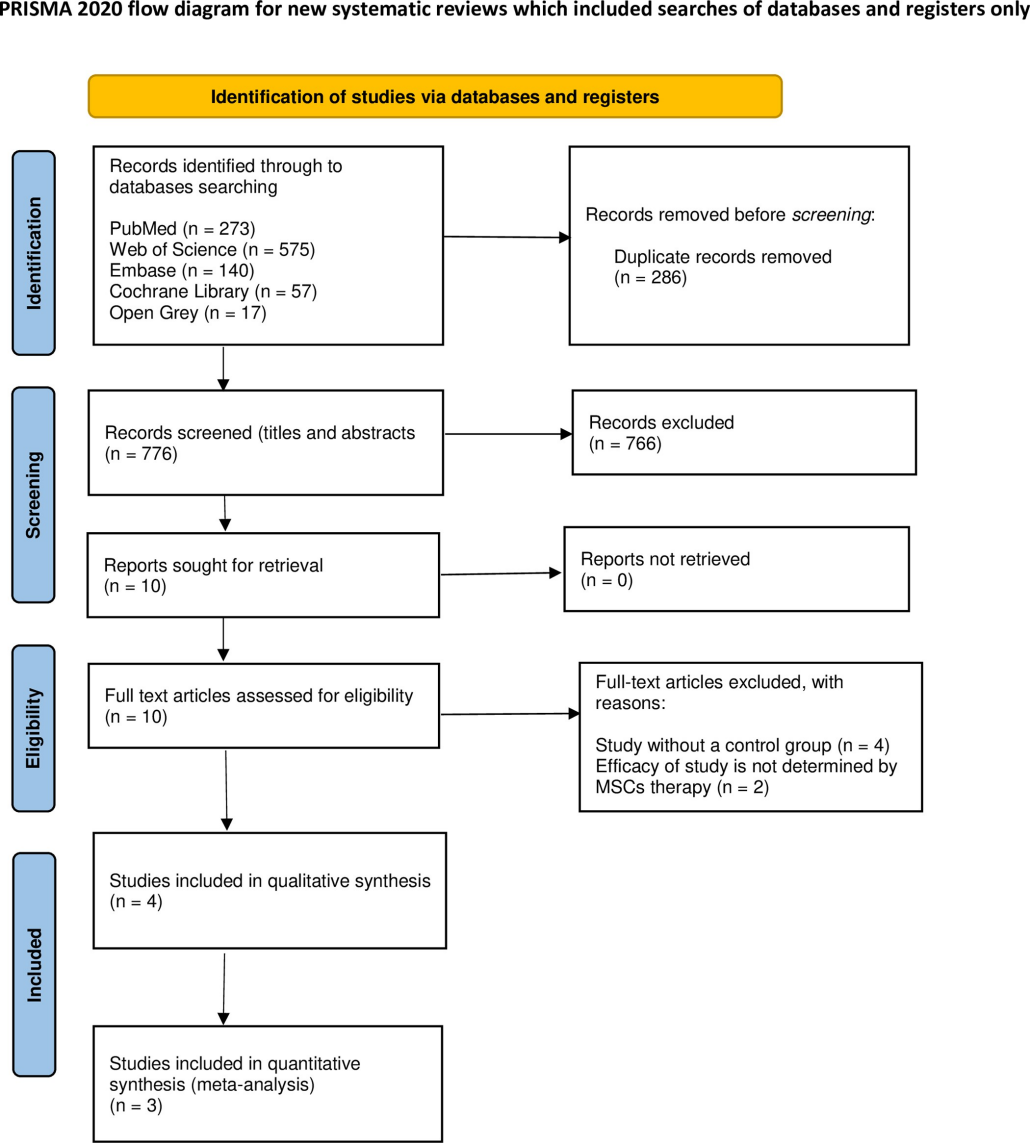
Flow diagram of the study identification process.

The systematic literature search identified 1062 records. We used an automated tool (Rayyan) to identify duplicate records in the eligibility process of this systematic review; after eliminating duplicates, 776 records remained. The records excluded after screening by title and abstract were 766, and only ten full-text articles were assessed for eligibility. Four studies without a control group and two studies where efficacy was not determined by MSCs therapy were excluded. Finally, four studies were included in this systematic review [6, 11, 13, 14], and three were used for quantitative synthesis [6, 11, 14]. Fig 1 shows the flow diagram of the study identification process.

### Study characteristics and quality assessment

**Fig 2:**
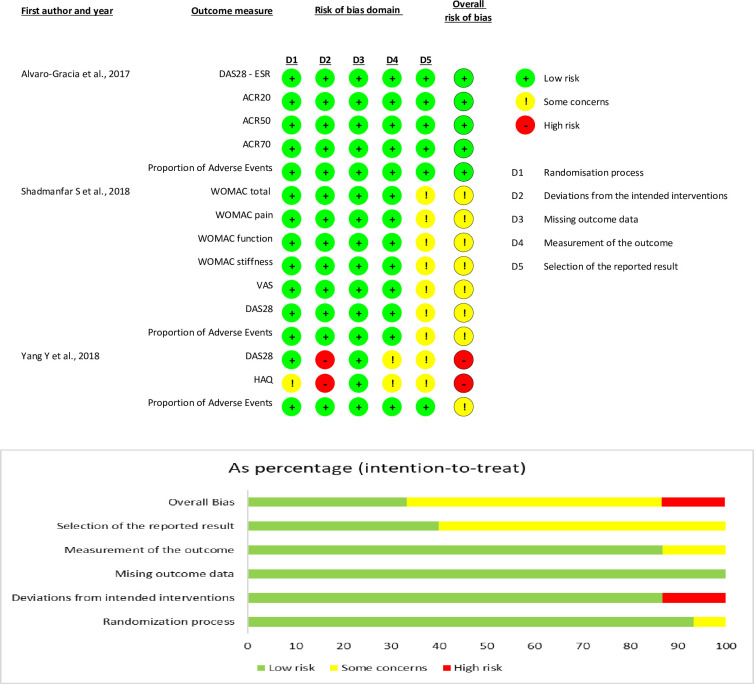
Risk of bias assessment of included RCTs–Cochrane RoB tool 2.0.

**Fig 3:**
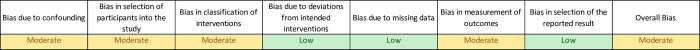
Risk of bias assessment in the non-RCTs of Wang, 2013 –ROBINS-I tool.

Four studies were included in this systematic review, three were RCTs, and one was non-RCTs. The included studies involved a total of 358 patients. The studies of Wang et al. (2013) and Yang et al. (2018) were conducted in China, Álvaro-Gracia et al. (2017) in Spain, and Shadmanfar et al. (2018) was conducted in Iran; only one study was multi-center (Álvaro-Gracia et al.). The study of Álvaro-Gracia et al. (2017) was divided into three subgroups according to the dosage, and the study of Yang et al. 2018 was divided into two subgroups according to the response or non-response of MSCs treatment, the route of administration was intravenous for 75% of the studies and intra-articular for 25% of these. The tissue sources of MSCs were: 50% umbilical cord (UC), 25% bone marrow (BM), and 25% adipose tissue (AT). Finally, the treatment assignments were masked in 50% of the studies. The included studies’ overall characteristics are shown in Table 1. Five (33.33%) out of 15 clinical outcome measures (one study) were assessed as having a high risk of bias; eight clinical outcome measures had some concerns (53.33%, 2 studies), and five clinical outcome measures (33.33%, 1 study) were judged to be at low risk of bias. The summary and graph of the risk of bias are shown in Figs 2 and 3.

**Table 1:** Overall characteristics of the included studies.

First author, year	Study population	Trial Registration number	Location	Sample Size	Intervention	Control	Dosage	Infusion route	Follow-up
Wang L et al., 2013	Patients with active refractory rheumatoid artrhritis	NCT01547091	China	172	UC-MSC + DMARDs	DMARDs + Medium without UC-MSCs	40 X 10^6^ UC-MSC	IV	Up to 12 months (48 weeks)
Álvaro-Gracia et al., 2017	Patients with active refractory rheumatoid artrhritis	NCT01663116	Spain	53	Ad-MSCs	Physiological saline	1x10^6^ Ad-MSCs/Kg 2x10^6^ Ad-MSCs/Kg 4x10^6^ Ad-MSCs/Kg	IV	Up to 6 months (24 weeks)
Shadmanfar S et al., 2018	Patients with rheumatoid arthritis who had knee involvement	NCT01873625	Iran	28	BM-MSCs	Physiological saline	42 ± 4 X 10^6^ BM-MSCs	IA	Up to 12 months (48 weeks)
Yang Y et al., 2018	Patients treated by active rheumatoid arthritis	ChiCTR-ONC-16008770	China	105	UC-MSCs	Physiological saline	1 X 10^6^ UC-MSCs/Kg	IV	Up to 12 months (48 weeks)

UC-MSCs, Umbilical Cord-derived Mesenchymal Stem Cells; DMARDs, Rheumatoid Arthritis with Disease-Modifying Drugs; Ad-MSCs, Adipose-derived Mesenchymal Stem Cells; BM-MSCs, Bone marrow-derived Mesenchymal Stem Cells; IV, Intravenous infusion; IA, Intra-articular injection.

### GRADE assessment

We use the GRADE approach to assess evidence for each important outcome and present the summary findings for all combinations of MSCs therapy and control interventions in the included RCTs and non-RCTs. These treatments were evaluated as clinical activity, measures of response by the ACR scale, and safety of therapy with MSCs. The grade of certainty of evidence for clinical activity outcome measures was considered high to low. The quality of evidence was downgraded for risk of bias, inconsistency, and imprecision of the results. Due to the few studies (n < 10) in the meta-analysis, the publication bias could not be excluded because the test power is usually too low to distinguish chance from real asymmetry. The certainty of evidence for DAS28-ESR outcomes was high (outcome measures were reported in one RCT). Certainty of the subscales of WOMAC as pain, function, stiffness outcomes was moderate (outcome measures were reported in one RCT); certainty for VAS, DAS28, and HAQ outcomes was high to low (outcome measures were reported in two RCTs). Certainty of evidence for measure of response outcomes as patients reaching ACR 20,50,70 was considered high to low (outcome measures were reported in two RCTs). Certainty of evidence for the safety of treatments: Adverse events (serious and non-serious adverse events) were low with inconsistency (1 level) and imprecision (1 level).

The evidence was downgraded for inconsistency and imprecision from 1 to 2 levels. None of the outcomes were upgraded based on risk of bias (see Table 2).

**Table 2:** GRADE assessment.

Comparison	Main outcome measures	No. of studies	Number of participants	Quality of evidence	Downgrading due to
Allogeneic Ad-MSCs vs Ringer’s lactate solution (Placebo)	*Clinical activity* Mean improvement of DAS28-ESR (4.9 points (95% CI: 4.104–5.696)) cohort A; (5.1 points (95% CI: 4.585–5.615)) cohort B; (2.0 points (95% CI: 1.265–2.735)) cohort C; (5.8 points (95% CI: 5.430–6.170)) placebo group. Three months after Ad-MSCs therapy. *Measure of response* Patients reaching ACR70 (5% cohort A; 0% cohort B; 16.7% cohort C; 0% placebo) three months after Ad-MSCs therapy. Patients reaching ACR50 (15% cohort A; 5% cohort B; 16.7% cohort C; 0% placebo) three months after Ad-MSCs therapy. Patients reaching ACR20 (25% cohort A; 15% cohort B; 16.7% cohort C; 0% placebo) three months after Ad-MSCs therapy.	1	20 (cohort A) 20 (cohort B) 6 (cohort C) 7 (placebo)	High	Study limitations (any limitations); Inconsistency (any inconsistency); Imprecision (the results were precise)
Autologous BM-MSCs vs Physiological saline	*Clinical activity* Mean change from baseline of WOMAC total in the BM-MSCs group -16.1 (-27.7–4.4) and placebo group -6.9 (-17.7–3.9) 12 months after BM-MSCs therapy (Effect size: 0.05; *p* = 0.25). Mean change from baseline of WOMAC pain in the BM-MSCs group -16.5 (-30.5–2.6) and placebo group -6.7 (-20.3–6.9) 12 months after BM-MSCs therapy (Effect size: 0.04; *p* = 0.31). Mean change from baseline of WOMAC function in the BM-MSCs group -16.5 (-30.4–2.6) and placebo group -9.6 (-20.8–1.6) 12 months after BM-MSCs therapy (Effect size: 0.02; *p* = 0.43). Mean change from baseline of WOMAC stiffness in BM-MSCs group -8.6 (-26.4–9.1) and placebo group 3.3 (-14.1–20.8) 12 months after BM-MSCs therapy (Effect size: 0.03; *p* = 0.33). Mean change from baseline of VAS in BM-MSCs group -2.2 (-3.6–0.9) and placebo group -1.7 (-4.0–0.6) 12 months after BM-MSCs therapy (Effect size: 0.005; *p* = 0.72). Mean change from baseline of DAS28 in BM-MSCs group -0.4 (-0.7–0.1) and placebo group -0.4 (-0.8–0.1) 12 months after BM-MSCs therapy (Effect size: 0.00008; *p* = 0.96).	1	28	Moderate	Imprecision (1 level)
Allogeneic UC-MSCs vs Physiological saline	*Clinical activity* Mean decrease of DAS28 in the Response group was -2.31 (95% CI or SD not reported); the No-Response group was -0.18 (95% CI or SD not reported), and the control group 0 (95% CI or SD not reported) 12 months after UC-MSCs therapy. Due to the authors presenting data as figures but not as tables, data were calculated with PlotDigitizer software from graphs. Mean decrease of HAQ in the Response group was -0.76 (95% CI or SD not reported); the No-Response group was 0.02 (95% CI or SD not reported), and the control group was -0.02 (95% CI or SD not reported) 12 months after UC-MSCs therapy. Data were calculated with PlotDigitizer software from graphs proportionated by the authors. Differences were not statistically significant between the No-Response group vs baseline; and the control group vs baseline.	1	105	Low	Imprecision (2 level)
MSCs (Ad-MSCs; BM-MSCs; UC-MSCs) vs Placebo	*Adverse Events (all studies)* Proportion of patients with adverse events in the MSCs group (183/111) and control group (38/75). *Serious Adverse Events (all studies)* Proportion of patients with serious adverse events in the MSCs group (3/111) and control group (0/75). *Non-serious Adverse Events (all studies)* Proportion of patients with non-serious adverse events in MSCs group (180/111) and control group (38/75).	3	186	Low	Inconsistency (1 level); Imprecision (1 level)
UC-MSCs + DMARDs vs DMARDs + Medium without UC-MSCs	*Measure of Response* Patients reaching ACR20 (33%); ACR50 (7%); ACR70 (7%) 8 months after the first UC-MSCs + DMARDs therapy	1	172	Low	Imprecision (2 level)

UC-MSCs, Umbilical Cord-derived Mesenchymal Stem Cells; Ad-MSCs, Adipose-derived Mesenchymal Stem Cells; BM-MSCs, Bone marrow-derived Mesenchymal Stem Cells; DMARDs, Rheumatoid Arthritis with Disease-Modifying Drugs; WOMAC, Western Ontario and McMaster Universities Osteoarthritis Index; VAS, Visual Analogue Scale; DAS28, Disease Activity Score-28; HAQ, Health Assessment Questionnaire; DAS28-ESR, Disease Activity Score-28 Erythrocyte Sedimentation Rate (ESR); ACR 20/50/70, Percentage of patients achieving ACR20/50/70 responses; CI, Confidence Interval; SD, Standard Deviation.

### Outcomes

#### Safety evaluation

**Fig 4:**
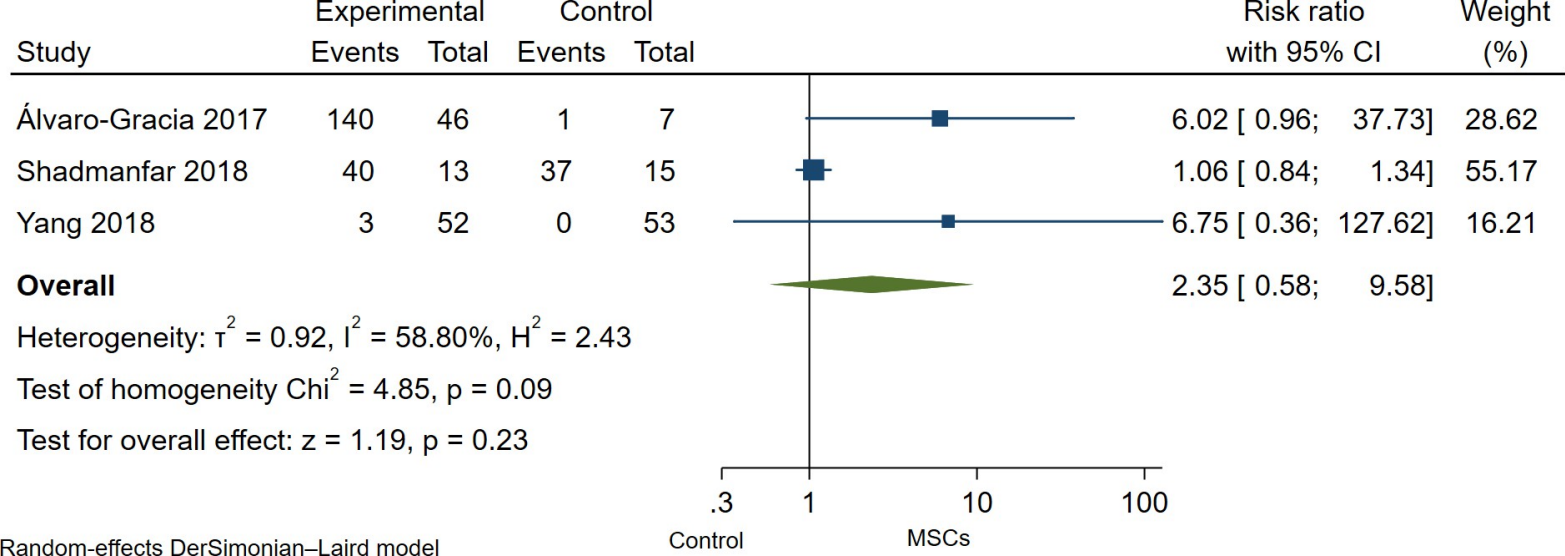
Forest Plot of the incidence of adverse events in the MSCs therapy group and control group.

**Fig 5:**
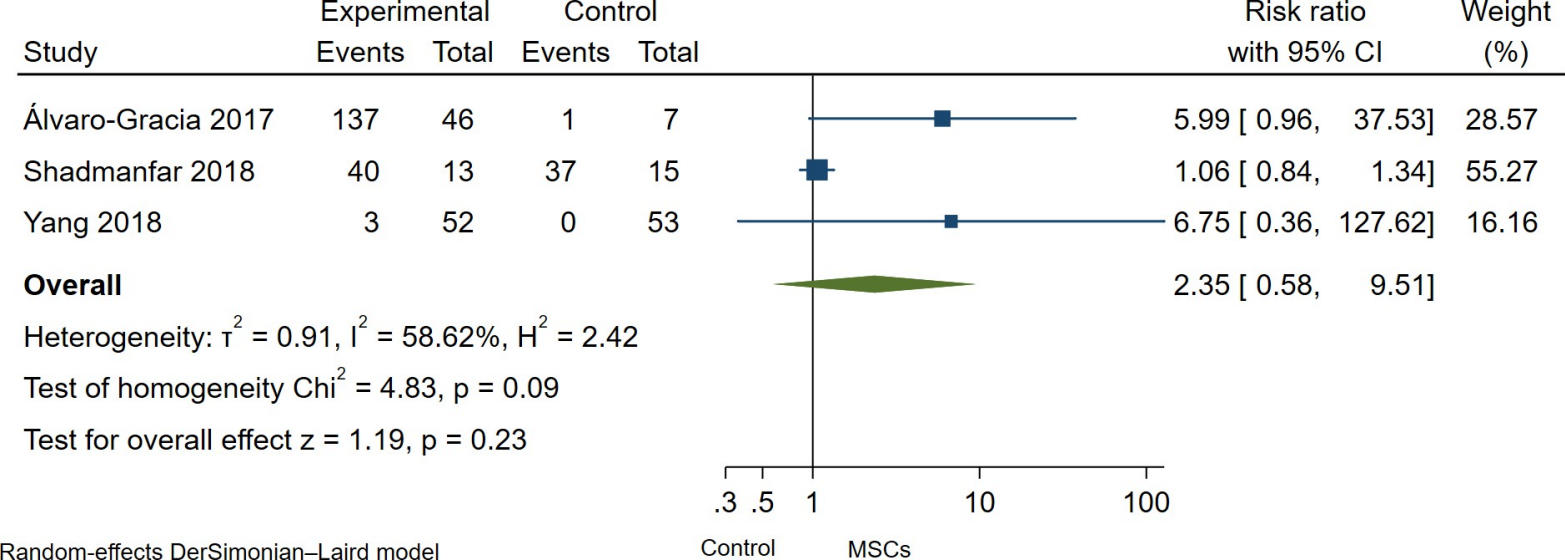
Forest Plot of the incidence of non-serious adverse events in MSCs therapy group and control group.

**Fig 6:**
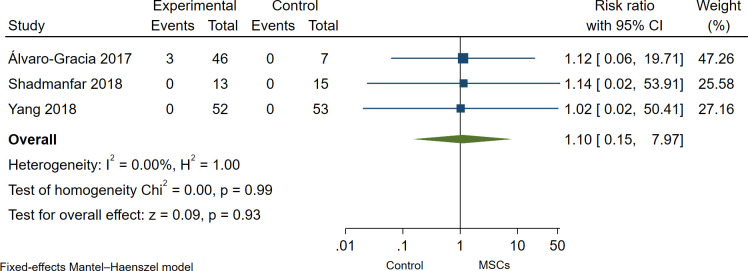
Forest Plot of the incidence of serious adverse events in MSCs therapy group and control group.

Overall, MSCs therapy was safe and well tolerated in all studies, as no serious adverse events (non-SAEs) were observed during or after MSCs administration. Wang et al. [13] reported mild adverse events such as chills and/or fever with two hours duration in 6 of 136 patients (4%); moderate and severe adverse events were not reported in this study, and death related to adverse events neither. Yang et al. [6] reported three patients with chills or fever (≤ 39°C) after MSC infusion with recovered within 3 hours; the authors reported non-serious adverse events. Furthermore, Shadmanfar et al. [14] found post-administration pain and/or articular swelling within one month after MSCs administration. However, patients responded to NSAIDs. Lastly, Álvaro-Gracia et al. [11] reported 141 adverse events (AEs) in 53 patients, 140 AEs in the MSCs group, and one AEs in the control group. Mainly AEs were fever, malaise, respiratory and urinary tract infection, ear infection, gastroenteritis, headache, nausea, vomiting, and diarrhea; 138 of 141 AEs were non-SAEs, and 3 were SAEs as lacunar infarction; peroneal nerve palsy and fever of moderate intensity. In the pooled analysis of RCTs [6, 11, 14], there was no statistical difference in the incidence of adverse events in the MSCs group as compared to the control group; however, the confidence intervals are wide in all effect sizes estimated; therefore, the effect-sizes estimates are imprecise, and it would be misleading to mention them without caution (Risk ratio: 2.35; 95% CI, 0.58 to 9.58; I^2^ = 58.80%; Fig 4). The pooled Risk ratio for non-serious and serious adverse events was no statistical difference in the incidence of non-serious and serious adverse events (Risk ratio: 2.35; 95% CI, 0.58 to 9.51; I^2^ = 58.62%; Fig 5) and (Risk ratio: 1.10; 95% CI, 0.15 to 7.97; I^2^ = 0.0%; Fig 6) respectively.

**Fig 7:**
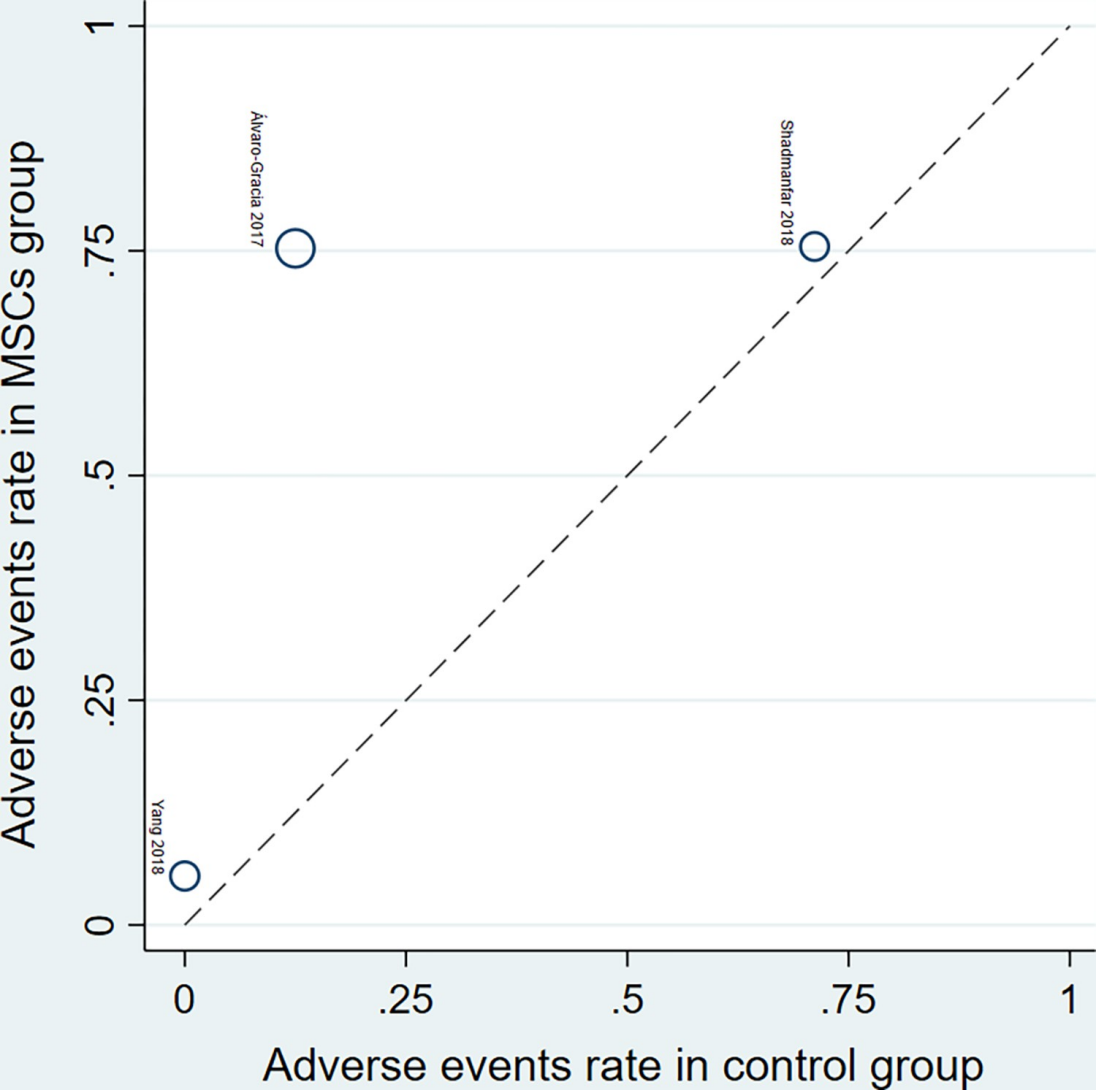
L’Abbé plot for the incidence of adverse events in the MSCs therapy group and control group.

In the L’Abbé plot for the rate of adverse events (Fig 7), each circle represents an individual trial, and larger circles represent trials with more adverse events; the dotted diagonal line indicates that the rate of adverse events is equal in the two arms within trials. In both the MSCs (treatment) and the control groups, it can be seen that the rate of adverse events varies greatly in both the MSCs groups (5% - 75%) and the control groups (0% - 71%) approximately. The rate of adverse events in the MSCs group was highest in the trial of Álvaro-Gracia et al. (75%). On the other hand, the rate of adverse events in the MSCs group was approximately the lowest in the trial of Yang et al. (5%).

Decreased total protein and globulin levels and increased albumin and hemoglobin levels after DMARDs plus UC-MSCs administration in comparison with the control group had shown in the study of Wang et al.; the function of the liver could be estimated based on this elevation in levels of albumin and hemoglobin and based on the decreased incidence of gastrointestinal tract bleeding. Álvaro-Gracia et al. not reported abnormal laboratory values nor relevant vital sign abnormalities, malignancies, or deaths. The study of Yang et al. was not found significant abnormalities in a routine blood test, liver, and kidney function analysis, urine analysis, or electrocardiography.

### Efficacy evaluation

Due to the great variability in evaluating clinical efficacy between studies included, it was impossible to perform a meta-analysis. The main differences between the RCTs were the following: a) Yang et al., the efficacy outcomes for the UC-MSCs group were evaluated according to the European League Against Rheumatism (EULAR) response criteria, which consider both the current DAS28 score and the reduction after treatment. Patients with a good or moderate response were allocated to the response group, and patients who had no response were allocated to the no-response group at 12 weeks after UC-MSCs treatment (the authors present data as figures but not as tables; therefore, data were calculated with PlotDigitizer software from graphs). On the other hand, Álvaro-Gracia et al., and Shadmanfar et al., allocated their RA patients to a UC-MSCs group and a placebo group. Data related to the number of applications and days of treatment of UC-MSCs therapy is not reported in the study of Yang et al. All studies reported the dose for delivery as a number of cells by kilogram of weight (MSCs/Kg), while Shadmanfar et al. reported the dosing as a number of cells by patient (MSCs/patient/dose). The efficacy outcome measures were different in all studies; Álvaro-Gracia et al. reported Patients’ EULAR Disease Activity Score (DAS28—Erythrocyte Sedimentation Rate (ESR)) at a follow-up frequency of 1, 2, and 3 months, while Yang et al., reported the 28-joint Disease Activity Score (DAS28); Shadmanfar et al. reported as disease activity index the Western Ontario and McMaster Universities Arthritis Index (WOMAC) at a follow-up frequency of 1,3,6 and 12 months. Finally, Yang et al. reported Patients’ EULAR Disease Activity Score (DAS-28) at follow-up frequency of 1, 2, and 3 weeks; 3, 6, and 12 months in the "Response UC-MSCs group" and "No-Response group." All efficacy outcome measures, follow-up periods, routes of administration, cell source, and dosing varied significantly between the three RCTs included. Despite the heterogeneity between efficacy measures, the main findings are summarized below.

The primary efficacy endpoints were the assessment of clinical activity, the measure of response: measured by the ACR scale (20, 50, 70), and serology.

*Clinical activity*. In the study of Wang et al., levels of HAQ (Health assessment questionnaire) and DAS28 (Disease Activity Score) scores decreased significantly three months after DMARDs plus UC-MSCs administration, indicating an improvement of clinical behaviors in comparison to the change in the scores of HAQ and DAS28 in the control group that was not significant (mean changes and 95% CI from baseline are not available in the published record); the authors reported a significant reduction of the number of joints with tenderness and swelling and relieving symptoms. Álvaro-Gracia et al. had shown clinical benefit in patients treated with adipose-derived stem cells (Ad-MSCs) as good EULAR response or low disease activity—erythrocyte sedimentation rate (DAS28-ESR <3.2); DAS28-ESR, through time, showed an overall decreasing tendency in Ad-MSCs cohorts; however, after three months, the clinical benefit did not persist in patients treated with Ad-MSCs. In Shadmanfar et al., the MSCs group showed improvements in WOMAC (Western Ontario and McMaster Universities Arthritis Index) pain score during the first month after treatment, which was maintained until the end of the trial; however, the improvement could not be significantly sustained after 12 months follow up (Effect size = 0.04; *p* = 0.31); Authors observed the same trend of effectiveness in WOMAC subscales and WOMAC total score (Effect size = 0.05; *p* = 0.25) in the BM-MSCs group versus placebo group. Although clinical activity comparisons between the BM-MSCs group and placebo group were not statistically significant differences, the BM-MSCs group had a better trend. Finally, Yang et al. found an improvement in the disease activity given that the HAQ and DAS28 scores of the response group were significantly decreased three months after MSCs administration (mean changes and 95% CI from baseline are not available in the published record), and most of the patients in the response group maintained these therapeutic effects for 12 months without continuous administration of MSCs.

*Measure of response in rheumatoid arthritis*. Wang et al. showed that 36% of patients (27 in 76) achieved ACR20, 28% (21 in 76) achieved ACR50, and 12% (9 in 76) achieved ACR70 in the MSCs group; in contrast with the control group where only 14% of patients (5 in 36) achieved ACR20. In group II (6 months after the first treatment), 47% (21 in 45) patients achieved ACR20, 20% (9 in 45) patients achieved ACR50, and 4% (2 in 45) achieved ACR70. Finally, in group III (8 months after the first treatment), 33% (5 in 15) patients achieved ACR20, 7% (1 in 15) patients achieved ACR50, and 7% (5 in 15) patients achieved ACR70. Álvaro-Gracia et al. evaluated the clinical efficacy between cohorts in an exploratory context, where responses for ACR20 in cohort A (1 x10^6^ Ad-MSCs/Kg) at months 1, 2, and 3 post-treatment were 45%, 25%, and 25%, respectively; In cohort B (2 x10^6^ Ad-MSCs/Kg), 20%, 30%, and 15%, respectively; In cohort C (4 x10^6^ Ad-MSCs/Kg), 33%, 17%, and 17%, respectively; in the placebo group (Ringer’s lactate solution), 29%, 14%, and 0%, respectively. In the intervention group, ACR50 responses tended to be greater than the placebo group, whereas ACR70 responses were very low. In the study of Yang et al., three months after MSCs administration, 54% of the patients in the MSCs group achieved a good or moderate response, whereas the other 46% of patients in the MSCs group had no clinical response.

*Serology*. Wang et al. showed decreased levels of CRP (C-Reactive Protein) and RF (Rheumatoid Factor) three months after DMARDs plus UC-MSCs treatment and increased levels of the percentage of CD4^+^, CD25^+^, and Foxp3^+^ (immunosuppressive) regulatory T cells in peripheral blood (mean changes and 95% CI from baseline are not available in the published record). In the study of Álvaro-Gracia et al., CRP evolution over time showed a general decreasing trend from baseline in all cohorts except in cohort B (2x10^6^ Ad-MSCs/Kg) and the placebo group. Shadmanfar et al. have not found significant changes in DAS 28 scores (Effect size = 0.00008; *p* = 0.96), ESR (Effect size = 0.00003; *p* = 0.97), and CRP (Effect size = 0.03; *p* = 0.37) between BM-MSCs and placebo groups. In the study of Yang et al., changes in levels of Anti-CCP (anti-cyclic citrullinated peptide antibody) and RF after treatment were not statistically significant (mean changes and 95% CI from baseline are not available in the published record); disease activity indices, such as ESR and CRP levels, decreased lightly to baseline levels, at the same time relapsed joint swelling, and pain.

### Sensitivity analyses

The small number of RCTs in each safety meta-analysis made it impossible the conduct sensitivity analyses.

### Publication bias

**Fig 8:**
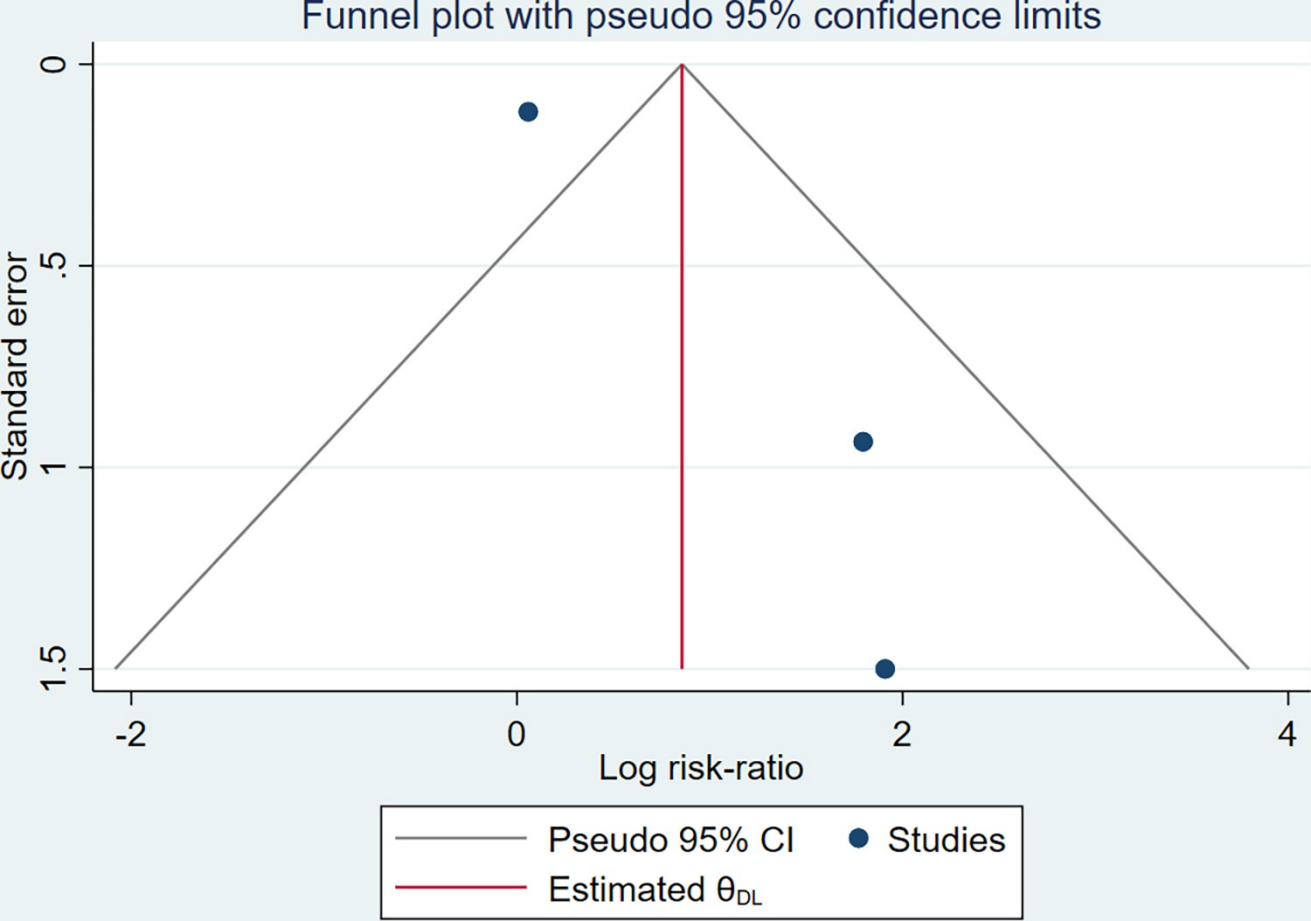
Funnel plot, using data from 3 trials of adverse events in MSCs therapy group and control group.

Publication bias was evaluated at the outcome level with the funnel plot (Fig 8), which showed some asymmetry. Given that few studies are in the meta-analysis, the test power is usually too low to distinguish chance from real asymmetry, and publication bias could not be excluded. When there is substantial heterogeneity, as in our case (I^2^ = 58.80%), the minimum number of studies recommended is more than 10 [15]. We also performed the Egger test of publication bias with *P* = 0.504 (> 0.05) and did not find significant publication bias. The unpublished information findings in ClinicalTrials.gov are shown in Table 3, complementing the information published to date.

**Table 3:** Summary of active RA clinical trials with MSC therapy that included a control group.

Clinical Trial Identifier	Registration Year	Country	Phase	Recruitment Status	Study Title	Condition	Intervention	Control	Outcomes	Sponsor	Estimated Study Completion Date
NCT04170426	2019	United States	Phase 1 Phase 2	Active, Not yet recruiting	Autologous Adipose-derived Stem Cells (AdMSCs) for Rheumatoid Arthritis	Refractory Rheumatoid Arthritis	Biological: autologous adipose derived stem cells	Placebo	Safety profile	Celltex Therapeutics Corporation	December 2025
NCT03186417	2017	United States	Phase 1	Recruiting	Mesenchymal Stem Cells in Early Rheumatoid Arthritis	Refractory Rheumatoid Arthritis	Biological: human Mesenchymal Stem Cells (hMSC)	Placebo	Safety profile and Preliminary Efficacy profile	MetroHealth Medical Center	May 2023
NCT03828344	2019	United States	Phase 1	Active, Not yet recruiting	Safety and Tolerability of a Single Intravenous Infusion of BX-U001 in Refractory Rheumatoid Arthritis	Refractory Rheumatoid Arthritis	Biological: Human Umbilical Cord Tissue Derived Mesenchymal Stem Cell (hUC-MSC)	Placebo	Safety profile and Preliminary Efficacy profile	Baylx Inc.	December 2024
ChiCTR1800018338	2018	China	Phase 1	Active, Not yet recruiting	A Clinical Trial for Human Gingiva Mesenchymal Stem Cells in the Treatment of Rheumatoid Arthritis	Refractory Rheumatoid Arthritis	Biological: methotrexate (MTX) conventional treatment + Gingival-derived Mesenchymal Stem Cells (GMSC)	methotrexate (MTX) conventional treatment + Placebo infusion	Safety profile and Preliminary Efficacy profile	Third Affiliated Hospital of Sun Yat-sen University	Unknown

## Discussion

Mesenchymal stem cell (MSC) therapies have been used as cell-based treatments during the last decades, owing to their potential applications such as anti-inflammatory, uncommon immunomodulatory competence, self-renewal, and multipotency properties. Many ongoing clinical trials are investigating the safety and efficacy of MSCs therapies to treat inflammatory diseases, which have shown positive clinical outcomes, with improved swollen joints, pain level, and quality of life. Furthermore, few RCT or non-RCT of MSCs conducted on rheumatoid arthritis (RA) patients have also shown some positive performance without serious adverse events. In this study, we presented a summary of active RA clinical trials with MSCs therapy that included a control group (Table 2), which will allow for establishing a more real efficacy and safety profile of MSCs required to investigate its applicability in clinical settings.

Our Systematic Review and Meta-analysis are the first work that substantially summarizes the safety of systemic MSCs administration isolated from the umbilical cord (UC), adipose tissue (AT), and bone marrow (BM) in the treatment of rheumatoid arthritis. This work did not find serious adverse events per system organ class, during or after MSCs administration, and provided initial safety and efficacy assessment for MSCs; in general, infusions of MSCs were safe and well tolerated in the included studies. Serum levels of cellular mediators that drive inflammation in RA, such as interleukin-6 and tumor necrosis factor alfa (TNF-α), decreased after the first MSCs treatment without evidence of toxicity at the dosage concentration in a period studied.

Due to the great variability in evaluating the clinical efficacy between the studies included, it was impossible to perform a meta-analysis for these outcomes; however, a comprehensive summary was performed, in which the HAQ and DAS28 scores decreased in agreement with the decreasing values of C-reactive protein (CRP) and Erythrocyte sedimentation rate (ESR). Additionally, a trend toward clinical efficacy was observed. However, studies did not show improvement beyond 12 months without continuous treatment administration.

Currently, the most well-known sources of MSCs are BM, AT, and UC; although MSCs can be isolated from any human tissue, there exist restrictions such as the availability of source tissues and invasiveness of the isolation procedures and different donor’s features [16] For instance, obtaining MSCs from BM can result in pain, excessive bleeding, infection of the skin at the site of the exam, and long-lasting discomfort, while that UC-MSCs exhibit outstanding characteristics such as self-renewal and multi-lineage differentiation abilities, moreover the sources for obtaining them, are easy to access and, with few law restrictions. Equally importantly, the less tumorigenic effect of UC-MSCs after transplantation is a remarkable finding [17]. Furthermore, UC-MSCs showed an outstanding inhibitory profile on serum levels of the IL-1α, IL-6, and IL-8 in lipopolysaccharides (LPS)-treated rats compared to AT-MSCs and BM-MSCs [16], given that a considerable number of active cytokines, such as TNF-α [18], IL-1 [19] and IL-6 [20] have been found in the joints of RA patients, which may produce joint damage, these immunoregulatory roles can reduce inflammation and serum levels of cellular mediators after administration of treatment and delay disease development like was evidenced in the clinical trials of Wang et al. [13] and Yang et al. [6] included in the present study.

## Conclusion

The evidence of effect is inconclusive for the efficacy outcomes, although the data showed a trend toward clinical efficacy. Our findings could suggest that MSCs therapy could be considered in treating RA when first-line treatment of RA as conventional synthetic disease-modifying anti-rheumatic drugs (cs-DMARDs) treatments have failed, and a biologic DMARDs is not feasible. Overall, a favorable safety profile and no life-threatening events were observed in subjects with RA. However, due to the methodological limitations of the included studies and the considerable heterogeneity between there, several high-quality, large sample sizes and extended follow-up RCTs should be performed to confirm the trend toward clinical efficacy observed in this study.

## Supporting information

S1 File PRISMA 2020 checklist. (DOCX)

S2 File Search strategies for each database. (DOCX)
