# Supplementary material for: Safety and efficacy of mesenchymal stem cells therapy in the treatment of rheumatoid arthritis disease: A systematic review and meta-analysis of clinical trials
Source: PLoS One. 2023 Jul 27;18(7):e0284828. doi: 10.1371/journal.pone.0284828 (PMC10374120; doi:10.1371/journal.pone.0284828)
Supplement: S2 File — (DOCX) [file pone.0284828.s002.docx]

**Search Strategy**

PubMed Search History (28/10/2022)

"(((((((((""arthritis, rheumatoid""[MeSH Terms]) OR (""rheumatoid arthritis""[Title/Abstract])) OR (""anti-citrullinated protein antibodies""[MeSH Terms])) OR (""anti-citrullinated protein antibodies""[Title/Abstract])) OR (""ACPAs""[Title/Abstract])) OR (""rheumatoid factor""[MeSH Terms])) OR (""rheumatoid factor""[Title/Abstract]) AND ((humans[Filter]) AND (english[Filter]))) AND (((((((""arthritis, rheumatoid""[MeSH Terms]) OR (""rheumatoid arthritis""[Title/Abstract])) OR (""anti-citrullinated protein antibodies""[MeSH Terms])) OR (""anti-citrullinated protein antibodies""[Title/Abstract])) OR (""ACPAs""[Title/Abstract])) OR (""rheumatoid factor""[MeSH Terms])) OR (""rheumatoid factor""[Title/Abstract]) AND ((humans[Filter]) AND (english[Filter])))) AND (((((((((((((((((((((((((((""Stem Cell Transplantation""[MeSH Terms]) OR (""Stem Cells""[MeSH Terms])) OR (""Stem Cell Research""[MeSH Terms])) OR (""Cell Therapy""[Title/Abstract])) OR (""cell based therapy""[Title/Abstract])) OR (""cell-based therapy""[Title/Abstract])) OR (""Stem Cell Transplantation""[Title/Abstract])) OR (""Multipotent Stem Cells""[Title/Abstract])) OR (""Multipotent Stromal Cells""[Title/Abstract])) OR (""Mesenchymal Stem Cells""[Title/Abstract])) OR (""Mesenchymal Stromal Cells""[Title/Abstract])) OR (""Mesenchymal Progenitor Cells""[Title/Abstract])) OR (""Wharton Jelly Cells""[Title/Abstract])) OR (""Wharton's Jelly Cells""[Title/Abstract])) OR (""Umbilical Cord Cell*""[Title/Abstract])) OR (""MSC""[Title/Abstract])) OR (""MSCs""[Title/Abstract])) OR (""ADMSC""[Title/Abstract])) OR (""ADMSCs""[Title/Abstract])) OR (""BM-MSC""[Title/Abstract])) OR (""BM-MSCs""[Title/Abstract])) OR (""BMD-MSC""[Title/Abstract])) OR (""BMD-MSCs""[Title/Abstract])) OR (""BMDMSC""[Title/Abstract])) OR (""BMDMSCs""[Title/Abstract])) NOT (""Hematopoietic Stem Cells""[Title/Abstract])) NOT (""mononuclear""[Title/Abstract]) AND ((humans[Filter]) AND (english[Filter])))) AND ((((((((""clinical""[Title/Abstract]) AND (""trial""[Title/Abstract])) OR (""clinical trials as topic""[MeSH Terms])) OR (""clinical trial""[Publication Type])) OR (""random*""[Title/Abstract])) OR (""random allocation""[MeSH Terms])) OR (""placebo""[Title/Abstract])) OR (""therapeutic use""[MeSH Subheading]) AND ((humans[Filter]) AND (english[Filter])))",,"Humans, English","((""arthritis, rheumatoid""[MeSH Terms] OR ""rheumatoid arthritis""[Title/Abstract] OR ""anti-citrullinated protein antibodies""[MeSH Terms] OR ""anti-citrullinated protein antibodies""[Title/Abstract] OR ""ACPAs""[Title/Abstract] OR ""rheumatoid factor""[MeSH Terms] OR ""rheumatoid factor""[Title/Abstract]) AND (""humans""[MeSH Terms] AND ""english""[Language]) AND ((""arthritis, rheumatoid""[MeSH Terms] OR ""rheumatoid arthritis""[Title/Abstract] OR ""anti-citrullinated protein antibodies""[MeSH Terms] OR ""anti-citrullinated protein antibodies""[Title/Abstract] OR ""ACPAs""[Title/Abstract] OR ""rheumatoid factor""[MeSH Terms] OR ""rheumatoid factor""[Title/Abstract]) AND (""humans""[MeSH Terms] AND ""english""[Language])) AND ((((""Stem Cell Transplantation""[MeSH Terms] OR ""Stem Cells""[MeSH Terms] OR ""Stem Cell Research""[MeSH Terms] OR ""Cell Therapy""[Title/Abstract] OR ""cell-based therapy""[Title/Abstract] OR ""cell-based therapy""[Title/Abstract] OR ""Stem Cell Transplantation""[Title/Abstract] OR ""Multipotent Stem Cells""[Title/Abstract] OR ""Multipotent Stromal Cells""[Title/Abstract] OR ""Mesenchymal Stem Cells""[Title/Abstract] OR ""Mesenchymal Stromal Cells""[Title/Abstract] OR ""Mesenchymal Progenitor Cells""[Title/Abstract] OR ""Wharton Jelly Cells""[Title/Abstract] OR ""Wharton's Jelly Cells""[Title/Abstract] OR ""umbilical cord cell*""[Title/Abstract] OR ""MSC""[Title/Abstract] OR ""MSCs""[Title/Abstract] OR ""ADMSC""[Title/Abstract] OR ""ADMSCs""[Title/Abstract] OR ""BM-MSC""[Title/Abstract] OR ""BM-MSCs""[Title/Abstract] OR ""BMD-MSC""[Title/Abstract] OR ""BMD-MSCs""[Title/Abstract] OR ""BMDMSC""[Title/Abstract] OR ""BMDMSCs""[Title/Abstract]) NOT ""Hematopoietic Stem Cells""[Title/Abstract]) NOT ""mononuclear""[Title/Abstract]) AND (""humans""[MeSH Terms] AND ""english""[Language])) AND (((""clinical""[Title/Abstract] AND ""trial""[Title/Abstract]) OR ""clinical trials as topic""[MeSH Terms] OR ""clinical trial""[Publication Type] OR ""random*""[Title/Abstract] OR ""random allocation""[MeSH Terms] OR ""placebo""[Title/Abstract] OR ""therapeutic use""[MeSH Subheading]) AND (""humans""[MeSH Terms] AND ""english""[Language]))) AND ((humans[Filter]) AND (english[Filter]))".

Web of Science 28/10/2022

1. (((((TS=("arthritis, rheumatoid")) OR TS=(rheumatoid arthritis)) OR TS=("anti-citrullinated

protein antibodies")) OR TS=("anti-citrullinated protein antibodies")) OR TS=("ACPAs")) OR

TS=("rheumatoid factor")

1. ((((((((((((((((((((((((((TS=("Stem Cell Transplantation")) OR TS=("Stem Cells")) OR TS=("Stem

Cell Research")) OR TS=("Cell Therapy")) OR TS=("cell based therapy")) OR TS=("cell-based

therapy")) OR TS=("Stem Cell Transplantation")) OR TS=("Multipotent Stem Cells")) OR

TS=("Multipotent Stromal Cells")) OR TS=("Mesenchymal Stem Cells")) OR TS=("Mesenchymal

Stromal Cells")) OR TS=("Mesenchymal Progenitor Cells")) OR TS=("Wharton Jelly Cells")) OR

TS=("Wharton's Jelly Cells")) OR TS=("Umbilical Cord Cell*")) OR TS=("MSC")) OR

TS=("MSCs")) OR TS=("ADMSC")) OR TS=("ADMSCs")) OR TS=("BM-MSC")) OR

TS=("BM-MSCs")) OR TS=("BMD-MSC")) OR TS=("BMD-MSCs")) OR TS=("BMDMSC")) OR

TS=("BMDMSCs")) NOT TS=("Hematopoietic Stem Cells" )) NOT TS=("mononuclear")

1. ((((((((TS=("clinical" )) AND TS=("trial" )) OR TS=("clinical trials as topic")) OR TS=("clinical

trial" )) OR SO=("clinical trial" )) OR TS=("random*" )) OR TS=("random allocation" )) OR

TS=("placebo")) OR TS=("therapeutic use" )

1. (TS=("humans")) OR TS=("patients")
2. #1 AND #2 AND #3 AND #4

EMBASE 28/10/2022

(('arthritis, rheumatoid':exp OR 'rheumatoid arthritis':exp OR 'anti-citrullinated protein

antibodies':exp OR 'anti-citrullinated protein antibodies':ti,ab,kw OR 'acpas':ti,ab,kw OR

'rheumatoid factor':exp OR 'rheumatoid factor':ti,ab,kw) AND [embase]/lim) AND (('stem

cell transplantation':exp OR 'stem cells':exp OR 'stem cell research':exp OR 'cell

therapy':ti,ab,kw OR 'cell based therapy':ti,ab,kw OR 'cell-based therapy':ti,ab,kw OR

'stem cell transplantation':ti,ab,kw OR 'multipotent stem cells':ti,ab,kw OR 'multipotent

stromal cells':ti,ab,kw OR 'mesenchymal stem cells':ti,ab,kw OR 'mesenchymal stromal

cells':ti,ab,kw OR 'mesenchymal progenitor cells':ti,ab,kw OR 'wharton jelly

cells':ti,ab,kw OR 'wharton* jelly cells':ti,ab,kw OR 'umbilical cord cell*':ti,ab,kw OR

'msc':ti,ab,kw OR 'mscs':ti,ab,kw OR 'admsc':ti,ab,kw OR 'admscs':ti,ab,kw OR 'bm-msc':ti,ab,kw OR 'bm-mscs':ti,ab,kw OR 'bmd-msc':ti,ab,kw OR 'bmd-mscs':ti,ab,kw OR

'bmdmsc':ti,ab,kw OR 'bmdmscs':ti,ab,kw) NOT 'hematopoietic stem cells':ti,ab,kw NOT

'mononuclear':ti,ab,kw) AND ('clinical':ti,ab,kw AND 'trial':ti,ab,kw OR 'clinical trials as

topic':exp OR 'clinical trial':it OR 'random*':ti,ab,kw OR 'random allocation':exp OR

'placebo':ti,ab,kw OR 'therapeutic use':kw,lnk) AND ('humans':exp OR 'humans':ti,ab,kw

OR 'patients':exp OR 'patients':ti,ab,kw) AND [english]/lim

Cochrane Library 28/10/2023

1 "arthritis, rheumatoid".sh. or "rheumatoid arthritis".ti,ab. or "anti-citrullinated protein antibodies".sh. or "anti-citrullinated protein antibodies".ti,ab. or "ACPAs".ti,ab. or "rheumatoid factor".sh. or "rheumatoid factor".ti,ab.

2 (("Stem Cell Transplantation" or "Stem Cells" or "Stem Cell Research").sh. or "Cell Therapy".ti,ab,tw. or "cell based therapy".ti,ab,tw. or "cell-based therapy".ti,ab,tw. or "Stem Cell Transplantation".ti,ab,tw. or "Multipotent Stem Cells".ti,ab,tw. or "Multipotent Stromal Cells".ti,ab,tw. or "Mesenchymal Stem Cells".ti,ab,tw. or "Mesenchymal Stromal Cells".ti,ab,tw. or "Mesenchymal Progenitor Cells".ti,ab,tw. or "Wharton Jelly Cells".ti,ab,tw. or "Wharton's Jelly Cells".ti,ab,tw. or "Umbilical Cord Cell*".ti,ab,tw. or "MSC".ti,ab,tw. or "MSCs".ti,ab,tw. or "ADMSC".ti,ab,tw. or "ADMSCs".ti,ab,tw. or "BM-MSC".ti,ab,tw. or "BM-MSCs".ti,ab,tw. or "BMD-MSC".ti,ab,tw. or "BMD-MSCs".ti,ab,tw. or "BMDMSC".ti,ab,tw. or "BMDMSCs".ti,ab,tw.) not "Hematopoietic Stem Cells".ti,ab,tw. not "mononuclear".ti,ab,tw.

3 ("clinical" and "trial").ti,ab. or "clinical trials as topic".sh. or "clinical trial".pt. or "random*".ti,ab. or "random allocation".sh. or "placebo".ti,ab. or "therapeutic use".sh.

4 "humans".sh. or "humans".tw. or "patients".sh. or patients.tw.

5 1 and 2 and 3 and 4
